# Uniparental isodisomy caused autosomal recessive diseases: NGS‐based analysis allows the concurrent detection of homogenous variants and copy‐neutral loss of heterozygosity

**DOI:** 10.1002/mgg3.945

**Published:** 2019-08-27

**Authors:** Bing Xiao, Lili Wang, Huili Liu, Yanjie Fan, Yan Xu, Yu Sun, Wenjuan Qiu

**Affiliations:** ^1^ Xinhua Hospital Shanghai Jiaotong University School of Medicine Shanghai China; ^2^ Shanghai Institute for Pediatric Research Shanghai China

**Keywords:** AGL, glycogen storage disease type‐III, leigh syndrome, SURF1, uniparental isodisomy

## Abstract

**Background:**

Uniparental disomy (UPD) leading to autosomal recessive (AR) diseases is rare. We found an unusual homozygous state in two nonconsanguineous families, and only one parent in each family was a heterozygote.

**Methods:**

Two patients with homozygosity for pathogenic variants were revealed by whole‐exome sequencing (WES), further Sanger sequencing found that only one of the parents was a heterozygote. Initial genotype and copy number variations analysis from WES data of probands involving whole chromosomes 1 and 9 containing these two pathogenic variants were performed, genome‐wide single‐nucleotide polymorphism (SNP) array analysis was used to confirm these results.

**Results:**

Whole‐exome sequencing identified a homozygous c.3423_3424delTG mutation in *AGL* in patient 1 and a homozygous c.241‐1G>C mutation in *SURF1* in patient 2. Further parental testing found that only the two patients’ healthy fathers were heterozygous. WES‐based copy number and genotype analysis found a copy‐neutral loss of heterozygosity (LOH) of whole chromosome 1 in patient 1 and of whole chromosomes 9 and 10 in patient 2. Further genome‐wide SNP array and family haplotype analyses confirmed whole paternal uniparental isodisomy (UPiD) 1 in patient 1 and paternal UPiD 9 and maternal UPiD 10 in patient 2. Therefore, UPiD caused AR monogenic glycogen storage disease type‐III (GSDIII) in patient 1 and Leigh syndrome in patient 2 through non‐Mendelian inheritance of two mutant copies of a gene from each patient's father.

**Conclusion:**

Our report highlights that a single NGS‐based analysis could allow us to find homozygous sequence variants and copy‐neutral LOH in such cases. Our report also describes the first case of GSDIII caused by UPiD 1 and Leigh syndrome caused by UPiD 9.

## 1 INTRODUCTION

Uniparental disomy (UPD) describes both copies of a chromosome pair inherited from only one parent. UPD is classified into uniparental isodisomy (UPiD) and uniparental heterodisomy (UPhD). UPiD refers to the inheritance of two copies of the same chromosome from one parent, while UPhD describes the inheritance of both homologous chromosomes from one parental pair (Liehr, 2010). UPD may not result in adverse effects on individuals; however, UPD can result in clinical conditions by producing either homozygosity for recessive mutations or aberrant patterns of imprinting in humans (Robinson, 2000). Both UPiD and UPhD could result in imprinting disorders, while only UPiD or UPiD/UPhD could result in autosomal recessive (AR) disorders through the inheritance of deleterious alleles from a carrier parent.

Recently, we encountered two patients with homozygosity for pathogenic variants revealed by whole‐exome sequencing (WES), and further parental testing found that only one of the parents was a heterozygote. Initial genotype analysis from WES data of probands revealed a copy‐neutral LOH region involving whole chromosomes 1 and 9 containing these two pathogenic variants, which were confirmed by single‐nucleotide polymorphism (SNP) array analysis. In a recent study, an approximately 1 Mb segmental copy‐neutral LOH region was detected by WES data analysis, which was verified by microarray analysis (Soler‐Palacín et al., 2018). WES mainly focuses on the detection of SNVs/indels in rare Mendelian diseases, and copy number variations (CNVs) analysis in the WES bioinformatics pipeline is increasingly common. Several reports using WES data to detect CNVs have been reported (D'Aurizio et al., 2016; Miyatake et al., 2015; Tan et al., 2014). In contrast, the use of WES data for LOH detection is limited (Bis et al., 2017; King et al., 2014; Soler‐Palacín et al., 2018), for the SNPs in WES are not pangenomic, and most SNPs with a high allele frequency are located in intronic regions, which limits their use in LOH detection. In this study, a single WES data analysis was initially performed to detect pathogenic variants, CNVs and LOH in two patients with the non‐Mendelian inheritance of two mutant copies of a gene from one parent.

## 2 MATERIALS AND METHODS

### 2.1 Patients

Patient 1 was a Han Chinese girl, and she was the only daughter of a nonconsanguineous couple. The family history was unremarkable. She was first noticed to have hepatomegaly at the age of 4 months. Liver biopsy was performed at 1 year and 6 months of age, and histological examinations showed glycogen accumulation. She came to our clinic at the age of 1 year and 8 months, and her mental development was delayed. Physical examination showed hepatomegaly, short stature, and muscle weakness. She had experienced episodes of hypoglycemia. Her echocardiogram was normal. Her blood biochemistry tests results were listed in Table 1.

**Table 1:** Blood biochemistry test results of patient 1

Parameters	Test value	Normal range
Creatine phosphokinase	587 U/L	26–192
LDH	605 U/L	106–211
Aspartate aminotransferase	439 U/L	8–38
Alanine aminotransferase	251 U/L	0–75
r‐GT	238 U/L	16–73
Uric acid	363 µmol/L	155–357
Fasting blood glucose	1.05 mmol/L	3.9–6.1
Triglycerides	3.38 mmol/L	0.2–2.31
Cholesterol	5.09 mmol/L	3.36–6.46
Lactate	1.2 mmol/L	0.7–2.1

Patient 2 was a girl who was the first child of healthy and nonconsanguineous parents. The family history was unremarkable. She was born following 32 weeks of pregnancy. Her birth weight was 880 g (<3rd). She has shown failure to thrive since birth. At 15 months old, psychomotor regression was observed. She showed a mildly delayed developmental profile for her chronological age based on the Gesell Developmental Schedules. The patient's bone age was 6 months. At 17 months old, aggravated vomiting was present, and she was admitted to the inpatient department of our hospital for hydrocephalus treatment at 18 months old. An operation was then performed, and an acute deterioration with seizures and vomiting emerged after the operation. Her height, weight, and head circumference at 18 months of age were 70 cm (<3rd), 8 kg (<3rd), and 48 cm (85th‐97th), respectively. Laboratory investigation revealed increased lactate (6.5 mmol/L) (normal range 0.7–2.1) and normal glycemia, creatinine kinase, amino acids, and organic acids. Computed tomography of the brain showed hydrocephalus. Her echocardiogram showed an atrial septal defect. Abdomen ultrasound showed normal results.

Informed consent was provided by the parents of both patients for a DNA study and the publication of clinical features. The study protocol was approved by the Ethical Review Board of Xin Hua Hospital (XHEC‐D‐2014–044).

### 2.2 Next‐generation sequencing

Genomic DNA was extracted from peripheral whole blood using a QIAamp DNA Blood Mini Kit (Qiagen) following standard procedures. The capture kits used for patient 1 and patient 2 were the xGen Exome Research Panel (Integrated DNA Technologies) and the SureSelect All Exon V5 (Agilent), respectively. WES was performed as previously described (Sun et al., 2017). We excluded variants from the candidate variant list that were observed with a frequency of >1% in the 1,000 Genomes Project, the Exome Aggregation Consortium (ExAC), or the Exome Variant Server (EVS) database with a frequency of >5% in a local database containing 1,000 exomes. Filtered variants of each patient were subsequently screened using autosomal recessive, autosomal dominant/de novo and X‐linked inheritance patterns. XHMM (Fromer et al., 2012) was applied to call CNVs in each sample. The calculation was performed for each patient with at least another 200 exomes captured by the same probe kit. Allele distribution was called for each high‐confidence SNP site from the 1,000 Genomes Project Phase 1 in probe regions. Variant allele frequency was plotted with CNV Kit (Talevich, Shain, Botton, & Bastian, 2016; https://doi.org/10.1371/journal.pcbi.1004873).

### 2.3 SNP arrays

The two patients were then analyzed with the Affymetrix CytoScan™ 750k (Santa Clara, California, USA). Genomic DNA was prepared from peripheral blood using the QIAamp DNA Blood Midi Kit (Qiagen). The SNP microarray experiment was performed using standardized protocols provided by the manufacturer. Affymetrix^®^ Chromosome Analysis Suite (ChAS) 1.2.2 (Affymetrix, Inc.) software was used to detect and analyze the patients’ chromosomal CNVs. The chromosome positions are shown according to GRCh 37 (hg19).

### 2.4 Haplotype analysis

To determine the parental origin of the UPD region of chromosome 1 in patient 1 and chromosomes 9 and 10 in patient 2, haplotyping was performed using the SNP genotype. The genotypes of the parents were then analyzed with Affymetrix CytoScan™ 750k (Santa Clara). The informative SNP markers within the UPD region were analyzed.

### 2.5 Sanger sequencing

Candidate variants were confirmed using Sanger sequencing. PCR primer sequences and protocols are available upon request. Amplified fragments were sequenced using a 96‐capillary 3730xl system (Applied Biosystems).

## 3 RESULTS

**Figure 1:**
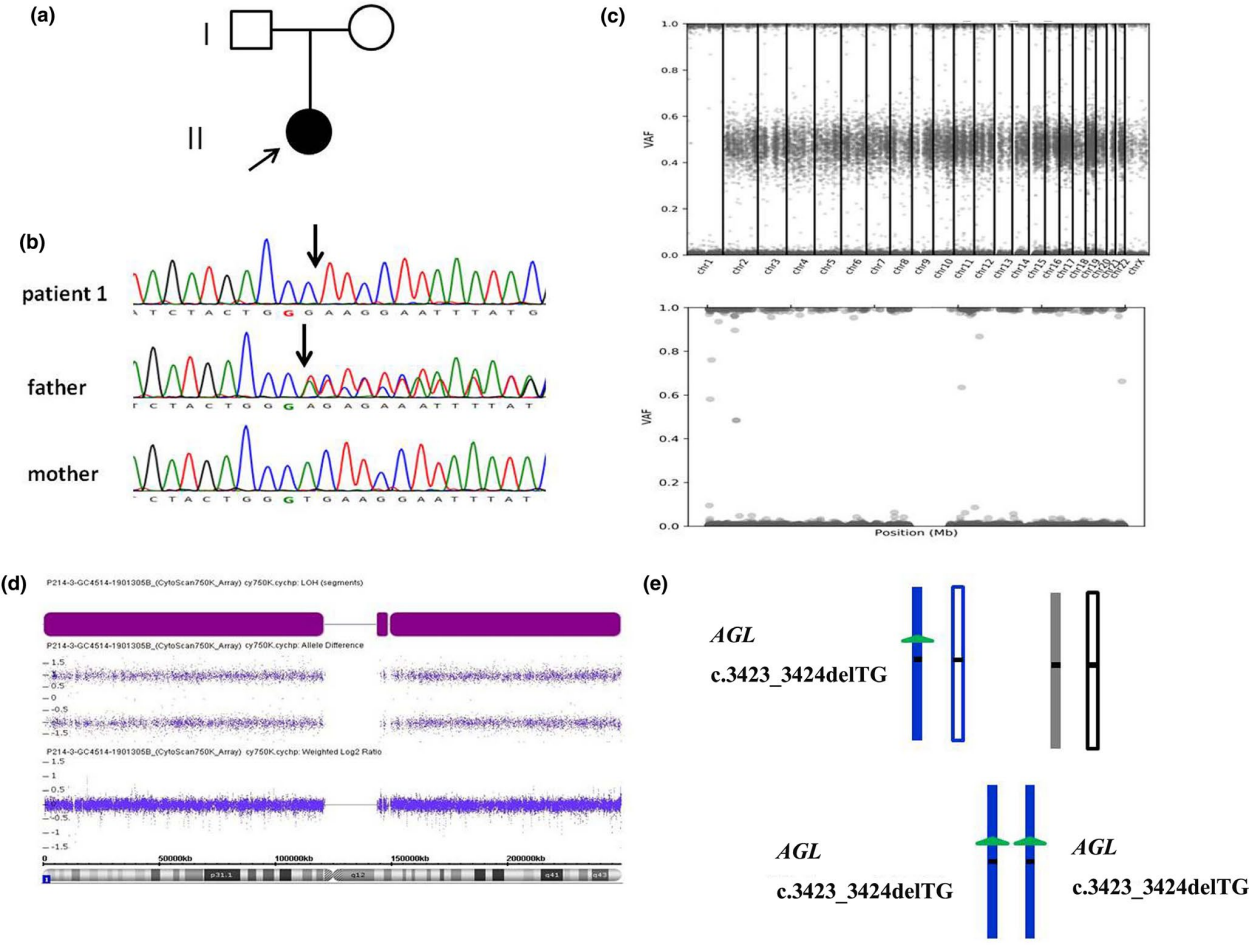
Whole UPiD 1 of paternal origin containing a homogenous *AGL* c.3423_3424delTG mutation in patient 1. (a) Family pedigree of patient 1. (b) Sanger sequencing of patient 1 and her parents: a homogenous *AGL* NM_000028.2: c.3423_3424delTG mutation was identified in the proband, and her father was heterozygous for the mutation. (c) The variant allele frequency plot of the genome (upper panel) and chromosome 1 (lower panel) from the exome sequencing data of patient 1. The *x* axis shows the position of each SNP site along the chromosome. The *y* axis represents the variant allele distributions. (d) Copy number and allele peak analyses of the SNP array in patient 1 and her parent. The allele peak analysis of patient 1 showed a 250 Mb LOH region in whole chromosome 1 (with a loss of the middle bands across entire chromosome 1); copy number panels revealed two copies of each gene on chromosome 1. Haplotype analysis using the SNP genotype from the array showed the paternal origin of whole chromosome 1. (e) Family of patient 1 shows the inherited chromosome 1 with the whole isodisomy of paternal origin

**Figure 2:**
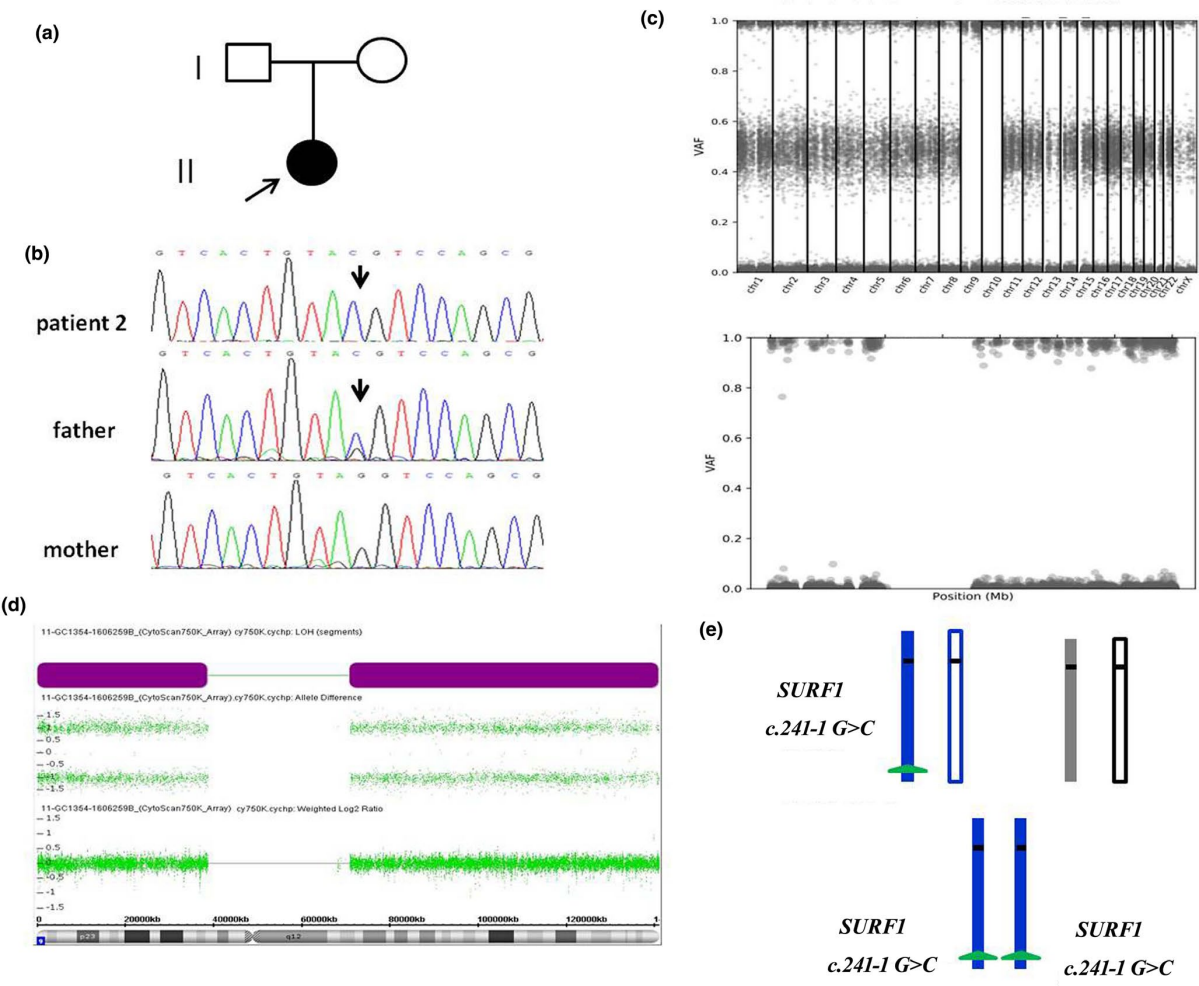
Whole UPiD 9 of paternal origin containing a homozygous *SURF1* c.241‐1G>C mutation in patient 2. (a) Family pedigree of patient 2. (b) Sanger sequencing of patient 2 and her parents: a homozygous *SURF1* NM_003172.3:c.241‐1G>C mutation was confirmed in patient 2, and her father was heterozygous for the mutation. (c) The variant allele frequency plot of the genome (upper panel) and chromosome 9 (lower panel) from the exome sequencing data of patient 2. The *x* axis shows the position of each SNP site along the chromosome. The *y* axis represents the variant allele distributions. (d) Copy number and allele peak analyses of the SNP array in patient 2 and her parent. The allele peak analysis of patient 2 showed a 141 Mb LOH region in whole chromosome 9 (with a loss of the middle bands across entire chromosome 9); copy number panels revealed two copies of each gene on chromosome 9, indicative of UPD. Haplotype analysis using the SNP genotype from the array showed the paternal origin of whole chromosome 9. (e) Family of patient 2 shows the inherited chromosome 9 with the whole isodisomy of paternal origin

Whole‐exome sequencing identified a homozygous *AGL* NM_000028.2: c.3423_3424delTG, p.Glu1142fs in patient 1 (Figure 1) and a homozygous *SURF1* NM_003172.3:c.241‐1G>C mutation in patient 2 (Figure 2). These two mutations were confirmed by Sanger sequencing and showed that both patients’ healthy fathers were heterozygous for the mutation and that their mothers did not carry the mutation. Considering the autosomal recessive inheritance of AGL and SURF1 deficiency, which were not consistent with the homozygous occurrence in two patients. These findings suggested the possibility of maternal chromosome 1 deletion involving the *AGL* locus or the possibility that the patient 1 inherited two copies of the mutant paternal *AGL* allele, and for the patient 2, either maternal chromosome 9 bore a deletion involving the *SURF1* locus or that the patient inherited two copies of the mutant paternal *SURF1* allele.

The patient 1's WES data showed an LOH region of whole chromosome 1 involving the *AGL* gene (Figure 1). The patient 2's WES data showed an LOH region of whole chromosome 9 involving the *SURF1* gene and LOH of whole chromosome 10 (Figure 2; Figure S1). SNP array and WES‐based copy number analyses of the two patients’ genomic DNA yielded normal results, thereby ruling out genomic copy number abnormalities.

A genome‐wide SNP array of the family 1 (the patient 1 and her parents) showed that the patient 1 was homozygous for a haplotype present in the father in the whole chromosome 1 region. We identified 50 common SNPs within the LOH region in which the patient 1 had not inherited any allele from her mother (Table S1). Collectively, these results indicate that the homozygous state of the patient 1's c.3423_3424delTG (p.Glu1142Argfs*24) mutation was due to paternal isodisomy of whole chromosome 1. A genome‐wide SNP array of the family 2 (the patient 2 and her parents) showed paternal isodisomy of whole chromosome 9, and the patient 2 was homozygous for a haplotype present in her father in the whole chromosome 9 region. Specifically, within the LOH region, we identified 30 SNPs in which the patient 2 had not inherited any allele from her mother (Table S2). Collectively, these results indicate that the homozygous state of the patient 2's c.241‐1G>C mutation was due to paternal isodisomy of chromosome 9. In addition, the SNP array of the family 2 showed maternal isodisomy of whole chromosome 10, and the patient 2 was homozygous for a haplotype present in her mother in the whole chromosome 10 region. We identified 31 SNPs within chromosome 10 in which the patient 2 had not inherited any allele from her father. Karyotype analysis of the patient 2 and their parent revealed no chromosomal aberrations involving chromosome 9 and 10.

These two mutations were not present in the 1,000 Genomes Project, gnomAD, EVS or in‐house databases, and c.3423_3424delTG mutation in patient 1 had previously been reported in the literature. According to the American College of Medical Genetics (ACMG) guidelines for variant interpretation (Richards et al., 2015), c.3423_3424delTG mutation could be classified as pathogenic (PVS1+PM2+PP4), and c.241‐1G>C mutation could be classified as pathogenic (PVS1+PM2+PP4). The clinical features and identified mutations observed in our two patients were submitted to the *AGL and SURF1* variant database (https://databases.lovd.nl/shared/individuals/AGL *or SURF1*) in the Leiden Open Variant Database (Fokkema et al., 2011).

## 4 DISCUSSION

We report an unusual homozygous state in two nonconsanguineous families and determined that only the fathers in these two families were heterozygous for the mutation. Such non‐Mendelian inheritance implied the possibility of gross deletion of the mother's allele, nonpaternity, UPD, or a de novo mutation (Tamura et al., 2015). WES and genome‐wide SNP array analyses excluded the deletion of the maternal allele, and family haplotype analysis using data from the SNP array confirmed that paternal isodisomy of whole chromosome 1 was present in patient 1 and that complete paternal isodisomy of chromosome 9 and maternal UPiD 10 were present in patient 2. Therefore, UPiD caused autosomal recessive GSDIII in patient 1 and Leigh syndrome in patient 2 through the non‐Mendelian inheritance of two mutant copies of a gene from the father.

GSDIII and Leigh syndrome are AR monogenic diseases that are always caused by the inheritance of two mutant alleles from each parent. All reported patients with a molecular diagnosis of these two disorders carry homozygous or compound heterozygous mutations. This is the first GSDIII patient and the first patient with Leigh syndrome caused by whole UPiD. GSDIII is caused by a deficiency in the glycogen debrancher enzyme and is associated with an accumulation of abnormal glycogen with short outer chains. It is characterized by hepatomegaly, hypoglycemia, and growth retardation. Muscle weakness in patients with IIIa is minimal in childhood but can become more severe in adulthood; some patients develop cardiomyopathy. From the clinical viewpoint, patient 1 presented classical clinical features of GSDIII and no other typical symptoms of GSDIII, suggesting no paternally imprinted genes on chromosome 1 that have a major effect on phenotype. Leigh syndrome is a rare, progressive neurodegenerative mitochondrial disorder of infancy with onset within the first months or years of life and may result in early death. Affected individuals usually show global developmental delay or developmental regression, hypotonia, ataxia, dystonia, ophthalmologic abnormalities, and classic findings on brain imaging. Biochemical studies in patients with Leigh syndrome tend to show increased lactate and abnormalities of mitochondrial oxidative phosphorylation. Clinical features of patient 2 showed typical features of Leigh disease and no other typical symptoms of Leigh syndrome, which also suggests that no paternally imprinted gene on chromosome 9 and no maternal chromosome 10 have a major effect on phenotype.

The incidence of UPD is estimated to be approximately 1:3500 live births (Robinson, 2000; Yamazawa, Ogata, & Ferguson‐Smith, 2010) and may not result in adverse effects on the individual if it does not involve chromosomes with imprinted regions. However, UPiD could lead to an AR disease through the inheritance of deleterious alleles from a carrier parent. UPiD in an AR disease was first reported in a cystic fibrosis patient caused by UPiD7 in 1988, which is thought to be a rare mechanistic basis for inherited disease (Spence et al., 1988). Similar cases with segmental or whole‐chromosome UPiD leading to recessive disorders were subsequently published that involved various chromosomes with different autosomal recessive diseases, in which whole UPiD accounted for 60% and segmental UPiD accounted for 40% (Niida, Ozaki, Shimizu, Ueno, & Tanaka, 2018). In the reported chromosomes with AR diseases, segmental or whole UPiD 1 is the most frequent, while UPiD 9 is rare; only two patients with segmental maternal UPiD 9, a patient with whole maternal UPiD 9 and a patient with complete paternal UPiD 9 have been reported, making our patient 2 the second paternal UPiD 9.

WES data have revealed the ability of single WES data to identify different types of genomic lesions, including SNVs, LOH, and CNVs. For these two patients described in this report, WES first revealed the disease‐causing mutations in the “homozygous” state inherited from one heterozygous parent. No deletion covering the region was detected by CNV analysis from the WES dataset. The LOH region involved in the whole chromosome was further detected by WES‐based analysis. Because LOH analysis from a WES dataset does not incur any additional costs, it is a bonus to incorporate LOH analysis in the WES bioinformatics pipeline, especially in cases with non‐Mendelian inheritance. The use of next‐generation sequencing (NGS) variant frequency data has allowed the detection of somatic LOH in tissue samples, and the results can be verified by microarray analysis (Margraf et al., 2017). A study applied the novel H^3^M^2^ algorithm to index patients’ WES data and successfully detected UPD events (Bis et al., 2017). And in another study, a novel method for detecting UPD from trio genotypes from SNP chip or WES data has developed and allowed for detection of pathogenic UPD events (King et al., 2014). These data demonstrate that the LOH region could be detected directly from the NGS data. However, small segmental LOH would be difficult to reveal due to the uneven spread of SNPs in WES. In a recent report, a patient's WES data identified the LOH region as small as 1.013 Mb in chromosome 4, which caused a homozygous mutation in the proband (Soler‐Palacín et al., 2018). More data are required to establish the size limitation of LOH detected by WES.

From these two cases and other similar cases in the literature, it is essential to determine the molecular basis of such diseases for genetic counseling of recurrent risk evaluation. UPD is mainly caused by a meiotic I/II error and/or postzygotic events when associated with trisomy rescue, monosomy rescue, or gamete complementation (Gardner & Sutherland, 2004). Whole UPiD is derived from an accidental error in meiosis, and segmental isodisomy is mainly considered the result of postzygotic mitotic recombination after normal fertilization (Niida et al., 2018). The recurrence risk for siblings is far less than 25%, as in general AR diseases. In patient 2 described herein, prenatal diagnosis was performed for her family in the second pregnancy, and the fetus was found to not inherit the mutant allele.

In summary, we reported the first glycogen storage disease type‐III (GSD‐III) patient with a reported homozygous mutation in *AGL* caused by whole UPiD 1 and the first Leigh syndrome patient with a novel homozygous mutation in *SURF1* caused by whole UPiD 9 through the non‐Mendelian inheritance of two mutant copies of a gene from the father. A comprehensive molecular analysis is necessary to identify UPD‐associated AR diseases in which the recurrence risk is low. A single NGS‐based analysis could allow us to find a homozygous sequence variant and copy‐neutral LOH in such cases.

## CONFLICT OF INTEREST

None declared.
