# Supplementary material for: Uniparental isodisomy caused autosomal recessive diseases: NGS‐based analysis allows the concurrent detection of homogenous variants and copy‐neutral loss of heterozygosity
Source: Mol Genet Genomic Med. 2019 Aug 27;7(10):e00945. doi: 10.1002/mgg3.945 (PMC6785455; doi:10.1002/mgg3.945)
Supplement: Supplementary file 1 [file MGG3-7-e00945-s001.docx]

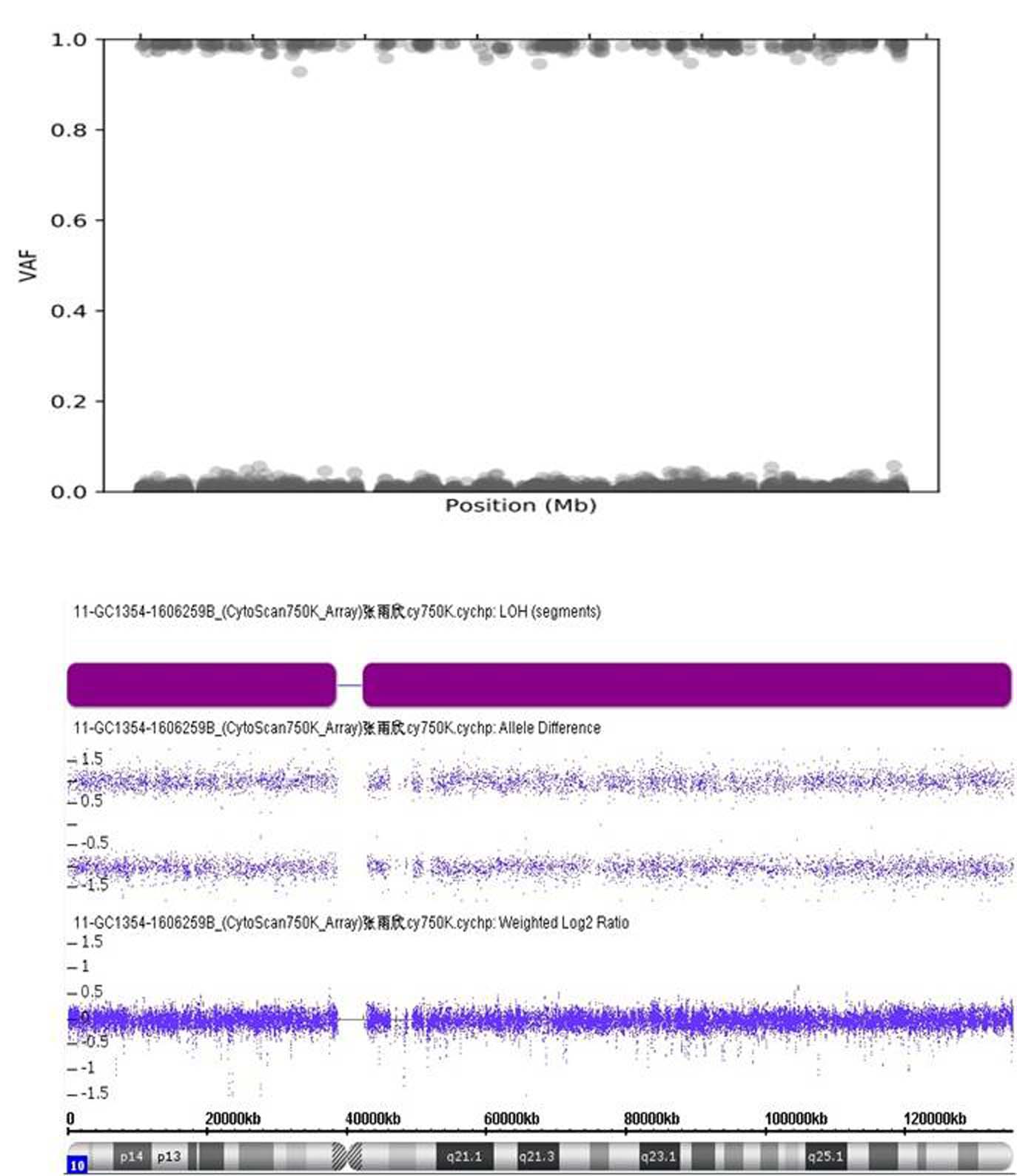


**Supplementary figure S1.** Whole UPiD 10 of maternal origin in the patient 2.

Upper: The variant allele frequency plot of chromosome 10 from exome sequencing data of patient 2. The x axis is the position of each SNP sites along the chromosome. The y axis represents the variant allele distributions. Lower: Copy number and allele peak analysis of SNP array in patient 2 and her parent. The allele peak analysis of the patient 2 showed a 135 Mb LOH region in whole chromosome 10 (with loss of the middle bands across entire chromosome 9), copy number panels reveal two copies of each gene on chromosome 10, indicative of UPD. Haplotype analysis using SNP genotype from array showed maternal orgin of whole chromosome 10.

**Supplementary table 1. Distribution of the informative SNP on chromosome 1 of the patient 1.**

| **SNP** | **chromosome** | **Location (hg19)** | **genotype** | | |
| --- | --- | --- | --- | --- | --- |
|  |  |  | patient 1 | father | mother |
| rs6696609 | 1 | 903426 | AA | AA | BB |
| rs10915683 | 1 | 4996185 | BB | AB | AA |
| rs2273299 | 1 | 10218439 | BB | BB | AA |
| rs6690379 | 1 | 14961661 | BB | AB | AA |
| rs16822735 | 1 | 20017831 | AA | AB | BB |
| rs7520844 | 1 | 25054223 | AA | AA | BB |
| rs271374 | 1 | 30124776 | AA | AA | BB |
| rs558969 | 1 | 35268693 | BB | AB | AA |
| rs12565115 | 1 | 40168587 | BB | AB | AA |
| rs59834392 | 1 | 45093761 | BB | BB | AA |
| rs7415684 | 1 | 52119198 | AA | AB | BB |
| rs1499663 | 1 | 56057990 | BB | AB | AA |
| rs10493278 | 1 | 60592321 | AA | AB | BB |
| rs6588096 | 1 | 65003469 | AA | AB | BB |
| rs2274913 | 1 | 70689215 | AA | AB | BB |
| rs7541725 | 1 | 75549806 | BB | AB | AA |
| rs299816 | 1 | 80415900 | AA | AA | BB |
| rs817482 | 1 | 85578057 | AA | AB | BB |
| rs9786929 | 1 | 90315408 | AA | AA | BB |
| rs10082010 | 1 | 95104632 | AA | AA | BB |
| rs35860087 | 1 | 97107135 | BB | BB | AA |
| rs1481505 | 1 | 99370353 | BB | AB | AA |
| rs10875223 | 1 | 99922216 | AA | AA | BB |
| rs4907906 | 1 | 100802399 | AA | AA | BB |
| rs4908074 | 1 | 101046092 | BB | AB | AA |
| rs11164417 | 1 | 102686309 | AA | AA | BB |
| rs10874636 | 1 | 103083831 | BB | AB | AA |
| rs12037024 | 1 | 105970509 | BB | AB | AA |
| rs3820664 | 1 | 110606081 | BB | AB | AA |
| rs4076194 | 1 | 115843927 | BB | AB | AA |
| rs10923916 | 1 | 120409703 | AA | AA | BB |
| rs7539416 | 1 | 146881586 | BB | BB | AA |
| rs11204791 | 1 | 151240542 | AA | AA | BB |
| rs56003102 | 1 | 156764917 | BB | BB | AA |
| rs2501873 | 1 | 161204538 | BB | BB | AA |
| rs1316805 | 1 | 166003309 | BB | AB | AA |
| rs183532 | 1 | 171609481 | AA | AA | BB |
| rs16849522 | 1 | 176016132 | AA | AB | BB |
| rs10494526 | 1 | 180279710 | BB | BB | AA |
| rs10798004 | 1 | 185265149 | BB | BB | AA |
| rs2990998 | 1 | 190118911 | AA | AA | BB |
| rs6682828 | 1 | 200507096 | AA | AA | BB |
| rs11240521 | 1 | 205533082 | AA | AA | BB |
| rs6678081 | 1 | 210121141 | BB | BB | AA |
| rs1436776 | 1 | 216235074 | AA | AA | BB |
| rs17011626 | 1 | 222702931 | BB | BB | AA |
| rs1938386 | 1 | 227097039 | BB | BB | AA |
| rs7534681 | 1 | 231908347 | BB | BB | AA |
| rs12130378 | 1 | 237277482 | BB | BB | AA |
| rs78540035 | 1 | 242005659 | AA | AA | BB |
| rs10436938 | 1 | 247706354 | BB | BB | AA |

**Supplementary table 2. Distribution of the informative SNP on chromosome 9 and 10 of the patient 2.**

| **SNP** | **chromosome** | **Location (hg19)** | **genotype** | | |
| --- | --- | --- | --- | --- | --- |
|  |  |  | patient 2 | father | mother |
| rs2360704 | 9 | 410312 | BB | BB | AA |
| rs4742091 | 9 | 559867 | BB | BB | AA |
| rs7869472 | 9 | 739736 | AA | AB | BB |
| rs7031845 | 9 | 1005131 | AA | AB | BB |
| rs10120924 | 9 | 2100016 | AA | AA | BB |
| rs41388145 | 9 | 3094840 | BB | AB | AA |
| rs6476798 | 9 | 4048636 | AA | AB | BB |
| rs10815144 | 9 | 5010192 | AA | AB | BB |
| rs10758734 | 9 | 6000551 | BB | AB | AA |
| rs818876 | 9 | 7007046 | BB | AB | AA |
| rs57526562 | 9 | 8075517 | BB | AB | AA |
| rs1333106 | 9 | 9307207 | AA | AB | BB |
| rs16925583 | 9 | 10236864 | BB | BB | AA |
| rs882123 | 9 | 20289153 | BB | AB | AA |
| rs62538326 | 9 | 30069805 | AA | AB | BB |
| rs1125350 | 9 | 71213397 | AA | AB | BB |
| rs965065 | 9 | 80387548 | BB | AB | AA |
| rs10868569 | 9 | 90002875 | AA | AB | BB |
| rs10981367 | 9 | 100017015 | AA | AB | BB |
| rs1538985 | 9 | 110131397 | AA | AB | BB |
| rs7866774 | 9 | 120082773 | AA | AA | BB |
| rs10987585 | 9 | 130036162 | BB | AB | AA |
| rs932887 | 9 | 135648905 | AA | AA | BB |
| rs10117024 | 9 | 135655027 | AA | AA | BB |
| rs11243967 | 9 | 135851114 | AA | AA | BB |
| rs11243968 | 9 | 135851156 | BB | BB | AA |
| rs11244065 | 9 | 136171412 | BB | BB | AA |
| rs1633759 | 9 | 136819288 | AA | AA | BB |
| rs10993895 | 9 | 136887726 | BB | AB | AA |
| rs465724 | 9 | 136910097 | AA | AB | BB |
| rs11537106 | 9 | 140319497 | AA | AA | BB |
| rs2246654 | 10 | 781124 | BB | AA | AB |
| rs2805562 | 10 | 1367514 | BB | AA | AB |
| rs2894969 | 10 | 4799607 | AA | BB | AB |
| rs3136627 | 10 | 5998285 | AA | BB | AA |
| rs10795586 | 10 | 8055876 | AA | BB | AB |
| rs11256641 | 10 | 10556916 | BB | AA | AB |
| rs11258687 | 10 | 13919458 | AA | BB | AB |
| rs10796292 | 10 | 15521848 | BB | AA | AB |
| rs11254543 | 10 | 17394523 | AA | BB | AA |
| rs2148299 | 10 | 20225654 | BB | AA | AB |
| rs60795529 | 10 | 25593723 | AA | BB | AB |
| rs1247098 | 10 | 30041106 | AA | BB | AB |
| rs1979442 | 10 | 36419573 | AA | BB | AA |
| rs1937989 | 10 | 42958204 | BB | AA | AB |
| rs11101084 | 10 | 50519197 | AA | BB | AB |
| rs7893174 | 10 | 55009632 | BB | AA | AB |
| rs10826267 | 10 | 60793767 | AA | BB | AA |
| rs1255463 | 10 | 65406161 | AA | BB | AB |
| rs3858147 | 10 | 70013264 | BB | AA | AB |
| rs7902668 | 10 | 76512725 | BB | AA | AB |
| rs4598621 | 10 | 80101868 | BB | AA | AB |
| rs1855954 | 10 | 85187593 | AA | BB | AB |
| rs4934411 | 10 | 90218338 | AA | BB | AA |
| rs10882199 | 10 | 95016790 | BB | AA | AB |
| rs11592082 | 10 | 100052464 | AA | BB | AB |
| rs1163081 | 10 | 105029840 | AA | BB | AB |
| rs10884631 | 10 | 110121247 | AA | BB | AB |
| rs17130192 | 10 | 114902990 | AA | BB | AB |
| rs236214 | 10 | 120122033 | AA | BB | AB |
| rs17105368 | 10 | 125250044 | BB | AA | AB |
| rs579655 | 10 | 130581094 | AA | BB | AA |
